# Unfolded p53 in the pathogenesis of Alzheimer's disease: is HIPK2 the link?

**DOI:** 10.18632/aging.100205

**Published:** 2010-09-22

**Authors:** Serena Stanga, Cristina Lanni, Stefano Govoni, Daniela Uberti, Gabriella D'Orazi, Marco Racchi

**Affiliations:** ^1^ Department of Experimental and Applied Pharmacology, Centre of Excellence in Applied Biology, University of Pavia, Italy; ^2^ Department of Biomedical Sciences and Biotechnologies, University of Brescia, Italy; ^3^ Department of Oncology and Experimental Medicine, School of Medicine, University “G. d'Annunzio”, 66100 Chieti, Italy; ^4^ Molecular Oncogenesis Laboratory, National Cancer Institute “Regina Elena”, 00158 Rome, Italy

**Keywords:** Alzheimer's disease, beta-amyloid peptides, p53 conformation, HIPK2

## Abstract

p53 transcriptional activity depends mainly on posttranslational modifications and protein/protein interaction. Another important mechanism that controls p53 function is its conformational stability since p53 is an intrinsically unstable protein. An altered conformational state of p53, independent from point mutations, has been reported in tissues from patients with Alzheimer's disease (AD), leading to an impaired and dysfunctional response to stressors. Recent evidence shows that one of the activators that induces p53 posttranslational modification and wild-type conformational stability is homeodomain interacting protein kinase 2 (HIPK2). Hence, conditions that induce HIPK2 deregulation would result in a dysfunctional response to stressors by affecting p53 activity. Discovering the mechanisms of HIPK2 activation/inhibition and the ways to manipulate HIPK2 activity are an interesting option to affect several biological pathways, including those underlying AD. Soluble beta-amyloid peptides have recently been involved in HIPK2 degradation, in turn regulating the p53 conformational state and vulnerability to a noxious stimulus, before triggering the amyloidogenic cascade. Here we discuss about these findings and the potential relevance of HIPK2 as a target for AD and highlight the existence of a novel amyloid-based mechanism in AD potentially leading to the survival of injured dysfunctional cells.

## INTRODUCTION

Alzheimer's disease (AD) is a primary progressive neurodegenerative disease where the aberrant meta-bolism of the amyloid precursor protein (APP) and the production and deposition of beta-amyloid peptide (Aβ) are considered responsible for neuronal death [1]. A putative link between the tumor suppressor p53 and the perturbation of APP metabolism has been demonstrated. In particular, an altered protein conformational state of p53, independent from point mutations, has been reported in tissues from patients with AD that led to an impaired and dysfunctional response to stressors [2-4]. One of the activators that induces p53 posttranslational modification and wild-type conformational stability is homeodomain-interacting protein kinase 2 (HIPK2) [5]. Here we discuss about the potential relevance of the definition of HIPK2 as a target for AD and highlight the existence of a novel amyloid-based pathogenetic mechanism in AD involving HIPK2 and unfolded p53, potentially leading to the survival of injured dysfunctional cells.

### Alzheimer's disease and beta-amyloid

**Figure 1.**
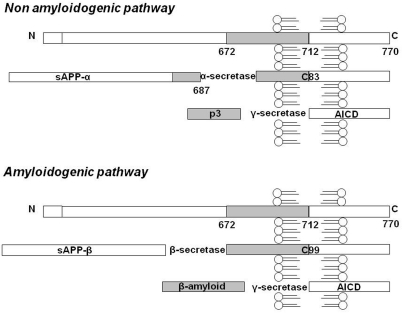
APP metabolism: schematic representation of the non-amyloidogenic and amyloidogenic pathway. Here the 770 residue APP processing is schematized, even if the 695 and 751 transmembrane forms of APP exist. In the non-amyloidogenic pathway, α-secretase cleaves APP in the extracellular domain and releases soluble APPα into the extracellular space. Following this cleavage, a second enzymatic product, the C-terminal fragment (αCTF or C83), which can be a substrate for ɣ-secretase, yields a non-amyloidogenic 3 kDa fragment known as p3. In the amyloidogenic pathway Aβ is formed following cleavage by β and ɣ secretases, respectively. The cleavage of APP at the residue 1 of Aβ sequence results in a truncated form of sAPP (sAPPβ) and in a C-terminal fragment of 12 kDa (βCTF or C99). The final step in the amyloidogenic pathway is the cleavage of βCTF, to liberate Aβ by ɣ-secretase. Furthermore, in both the amyloidogenic and non-amyloidogenic pathways, the cleavage of C83 and C99 fragments by ɣ-secretase also results in the generation of C-terminal peptides of 57-58 residues, referred as APP intracellular domain (AICD).

Beta-amyloid in AD is the result of the proteolytic metabolism of APP, an integral cell membrane glycoprotein of 697-770 residues which is the substrate of three proteolytic enzymes in two alternative pathways mutually in equilibrium [6]. In the non-amyloidogenic pathway, a protease, named α-secretase, cleaves APP in the extracellular domain and releases the ectodomain of APP (soluble APPα) into the extracellular space, thus precluding Aβ formation. Otherwise, in the amyloidogenic pathway, Aβ is formed following cleavage by β and ɣ secretases, that cleave the N and C terminus of Aβ, respectively (Figure 1). The two main isoforms found in AD brains are Aβ 1-40 and Aβ 1-42. Physiologically the 40-amino-acid long peptide is the most abundant form [7-9], since the concentration of secreted Aβ 1-42 is about 10% that of Aβ 1-40 [10]. For these reasons, Aβ1-40 and Aβ1-42 may have different biological actions [11] and the ratio of their production may be altered in pathological conditions, such as in familial AD [12].

Although the direct and the indirect neurotoxic role of Aβ are unchallenged (for an exhaustive review see [13]), recent findings suggest that the peptide may have so far unforeseen physiological roles [14]. Besides its presence in AD brains, experimental evidence indicated that Aβ peptides are produced constitutively by all cells, including neurons, and are found in the nM-pM range in the CSF of non-demented individuals [15] and in media from neuronal and non-neuronal cell cultures [16,17], thus suggesting that, as well as having a potential pathological role in AD, Aβ peptides under normal conditions may have a role in the regulation of physiological functions, consistent with their ubiquitous presence and normal synthesis. We will discuss here on how Aβ may have a role in the regulation of the function of p53.

### p53 function and its role in aging and neurodegeneration

From its discovery in 1979 [18], p53 continues to fascinate scientists and it is still nowadays one of the most extensively studied protein. Such interest is due to the key role of p53 in the prevention of cancer so as to be defined the "guardian of the genome" [19]. However, activities of p53 might have a role not only in regulating cancer progression, but also in the control of other aspects of health and disease such as development, aging and metabolism [20]. p53 exerts its main biological role of tumor suppressor and master controller of the genomic integrity especially acting as transcription factor [21]. p53 oversees the correct implementation of processes and it intervenes only in case of dangerous deviations from the proper cellular activity. When the cell is exposed to critical conditions or undergoes damages p53 arbitrates cell faith [22]. Loss of p53 or deregulation of its activities leads not only to cancer but also to cardiovascular, metabolic diseases, neurodegeneration and to the process of aging, because of the great number of p53-regulated genes which underlie all these different biological events [23] (see Table 1).

**Table 1.** p53 at the crossroad of complex networks of stress response pathways Different intercellular and extracellular stresses result in cellular outcomes directly mediated by p53 activation. The activation of p53 passes through a variety of modifications that occur at the protein level; these post-translational modifications are crucial in regulating p53 function. We summarize in the table p53 signalling transduction pathways resulting in activation of specific downstream gene targets, whose role is to drive cell destiny.

Cellular outcome	p53 gene target	Cellular stress	Molecule modifier	p53 residue and type of modification	Reference
**Cell cycle**	p21	Mild DNA damage	PCAF	Lys320-Acetylation	24
**checkpoint**	Gadd45	Mild DNA damage	E4F1	Lys320-Ubiquitylation	25
		UV radiation	CK2	Ser392-Phosphorylation	26, 27

**Apoptosis**	Transactivation: Bax, Bcl-X1, Apaf-1, Fas, Bad, Noxa, Puma	Severe DNA damage	HIPK2	Ser46-Phosphorylation	28, 29, 30, 31
		Severe DNA damage	CBP	Lys382-Acetylation	28
		UV radiation	MAPK	Ser46-Phosphorylation	32
	Transrepression: Bcl-2, Bcl-X1, Survivin	Genotoxic stress	DYRK2	Ser46-Phosphorylation	33
		Genotoxic stress	PKCδ	Ser46-Phosphorylation	34
		Severe DNA damage	MOF and TIP60	Lys120-Acetylation	35, 36
		Severe DNA damage	p300	Ls373-Acetylation	37

**Senescence**	p21	DNA damage	PML IV	Lys382-Phosphorylation	38
	p66	DNA damage	PML IV	Ser20-Phosphorylation	38
		Senescence stresses	ATM/Chk2	Ser15, Ser20-Phosphorylation	32, 39
		Senescence stresses	ATR/Chk1	Ser15, Ser37-Phosphorylation	32, 39, 40

The very first signal which stimulates p53 activity is the DNA damage and various genotoxic insults that could constitute a danger to the genomic integrity of cells such as oxidative stress, DNA damage, hypoxia, oncogene activation, telomere erosion, changes in metabolism, unusual prolongation of some signaling pathways and local depletion of nutrients, among others [41]. Immediately after these damages p53 is the substrate of a great number of possible post-translational modifications introduced by a variety of specific enzyme systems [42]. These alterations comprise a large network of covalent changes inducing characteristic modifications within the protein quantity, activity and ability to interact or cooperate with a variety of other proteins [43]. The delicate balance between p53 Structure and ActivityRelationship (SAR) can be disrupted even by a single amino acid substitution within the DNA binding domain (DBD) which is sufficient to limit or abolish the capacity of p53 to direct sequence-specific transcriptional activity [44]. This is the case of the majority of human cancers in which missense mutations in the DBD result in an altered network which can affect the prognosis. Beside gene mutation, p53 activity could be impaired also as consequence to a conformational change. p53 may lose its transcriptional activity due to an unfolded tertiary structure which determines a reduction in its affinity for specific DNA target sequence. Recent observations confirm that p53 structure changes can play a central role in aging and in AD [45,46].

Because of its role in establishing senescence and in determining organism aging when its activity is increased, p53 can promote selected aspects of the aging process. Different studies indicate a delicate balance between the tumor suppressive and age-promoting functions of p53. In several mouse models and also from human population studies, alteration of p53 activity has been demonstrated to influence the comparison of premature/accelerate aging under some circumstances (such as stress) or otherwise induce tumor suppression [47-52]. Evidence in mice and humans suggest that p53 acts as a longevity-assurance gene, basically reducing the influence of tumorigenesis [53,54].Our group has studied p53 in fibroblasts from aged controls and demented patients finding that with aging there is an increase in the expression of an unfolded protein state, which is more pronounced in AD patients and is not dependent on gene mutations [55]. As a result of such conformational change, p53 partially loses its activity and shows a significant impairment in its DNA binding and transcriptional capacity when cells are exposed to a noxious stimulus [55]. In fact, AD fibroblasts are less vulnerable to oxidative injury than fibroblasts from non-AD subjects to the point that conformationally altered p53 has been proposed as putative biomarker for early AD [55]. This altered conformation can be due to a loss of zinc (Zn^2+^) ion in the core domain of the protein, that provides the basic scaffold for the DNA binding and which has been demonstrated to be crucial for the stabilization of p53 in the so called "wild-type" folded form. Exposure of wild-type p53 (wt53) to metal chelators such as ethylenediaminetetraacetic acid (EDTA) or orthophenanthroline determines a rapid switch to the unfolded form positive to an antibody (clone PAb240), recognizing a primary epitope cryptic in wt53 [56]. Upon addiction of micromolar amounts of Zn^2+^, the protein undergoes a refolding to the native form and reacquires DNA-binding competence [56].Trying to investigate the cause of this alteration we found that the exposure to nanomolar concentrations of the beta-amyloid peptide 1-40 (Aβ 1-40) induced the expression of the unfolded p53 protein isoform in fibroblasts derived from non-AD subjects [3]. These data suggested that the tertiary structure of p53 and the sensitivity to p53-dependent apoptosis is influenced by low concentrations of soluble Aβ. On this basis, we assumed that low amounts of soluble Aβ induce early pathological changes at cellular level that may precede the amyloidogenic cascade and one of these changes is the induction of the unfolded state of p53, suggesting a role of the protein in the early pathogenesis of AD [3]. Recently, in cultured peripheral blood cells derived from AD patients we and others observed a detectable amount of unfolded p53, recognized with the antibody PAb240, which made these cells distinct from those of controls. We suggest that unfolded p53 could be used as a biomarker of the disease also in early stages [57,58]. Zhou and collaborators speculate that unfolded p53 might be the responsible for the failure of G1/S transition checkpoint in AD lymphocytes, which is normally mediated by wt53, connecting unfolded p53 to a peripheral event associated to the disease. They suggest that the cause of p53 conformational change could be oxidative stress, Aβ toxicity and the effects of oxygen free radicals [59]. This additional observation about the existence of an altered state of p53 at the peripheral level in subjects with AD reinforces the hypothesis that the protein can have a role in the pathogenesis of the neurodegenerative disease. However, further studies are needed to understand the causes of such conformational change and, as consequence, about how unfolded p53 contributes to the progress of age and neurodegeneration.

### Misfolded p53 and the role of HIPK2

Homeodomain-interacting protein kinase 2 (HIPK2) is a member of a novel family of nuclear serine/threonine kinases that localizes into the nuclear bodies and acts as co-repressor for several transcription factors [60]. Furthermore, one important function of HIPK2 is the apoptotic activation of p53 in response to genotoxic agents [5]. HIPK2 interacts physically and functionally with p53 phosphorylating it at Serine 46 (Ser46) for apoptotic activation (Table 1) [28-31]. HIPK2 interacts also with the acetyltransferase CREB binding protein (CBP) and co-localizes with CBP and p53 at promyelocytic leukemia nuclear bodies (PML-NBs); here HIPK2-mediated p53Ser46 phosphorylation enhances CBP-mediated p53 acetylation at Lys382, potentiating the expression of pro-apoptotic target genes [28].Thus, although Ser46 can be phosphorylated by additional kinase other than HIPK2, including ATM [61], DNA-dependent protein kinase (DNA-PK) [62], protein kinase C δ (PKCδ) [63], and dual-specificity tyrosine-phosphorylation-regulated kinase 2 (DYRK2) [64], the fact that only HIPK2 can drive Lys382 acetylation renders this kinase a unique and complex regulator of p53 apoptotic function. Thus, in the absence of HIPK2, the lack of Lys382 acetylation strongly impairs p53 pro-apoptotic activation [65]. HIPK2 function is important for the p53 acetylation/deacetylation balance by regulating the activity of deacetylase Sirt1, through repression of NADPH oxidase 1 (Nox1) [66]. Thus, in the absence of HIPK2, oxygen reactive species (ROS) are induced in cancer cells, with activation of Nox1 and Sirt1 activities that inhibit p53 apoptotic activity in response to DNA damage [66].

The role of HIPK2 in p53 activation in cancer cells involves also wt53 protein conformation. In the absence of HIPK2, p53 acquires a misfolded conformation loosing DNA binding and transcriptional activities, depending on deregulation of metallothioneins and Zn^2+^ [67,68]. Thus, Zn^2+^ supplementation to HIPK2 depleted cancer cells determines a regain of the wt53 protein conformation and restoration of DNA binding and transcriptional activities in response to genotoxic agents *in vitro* and *in vivo* [67]. Treatment of mice carrying tumors derived from HIPK2-depleted cells with a combination of Zn^2+^ and chemotherapeutic drug Adriamycin enhances growth suppression of such tumors *in vivo*[67]. From these data it appears that HIPK2 plays a major role in the regulation of p53 function through the switch between p53 dynamic conformational states, and that Zn^2+^ is a fundamental cofactor.

The binding and exchange/transport of Zn^2+^, as well as of other heavy metals, such as cadmium or copper, are modulated by metallothionein (MT), a family of at least 10 highly conserved, low molecular weight cystein-rich metalloproteins [69]. The interest in MTs derives from their role as regulators of p53 folding and activity, since small amount of MTs can induce p53 activity regulating the folding of the DBD domain through Zn^2+^ modulation, whereas excess of MTs reduces p53 activity by exerting their Zn^2+^ chelator function [70,71].Furthermore, an increase of MTs expression also correlates with chemoresistance, increased cell proliferation, reduced apoptosis and inhibition of p53 activity in various human tumors [72]. In this regard, it has been shown that HIPK2 negatively regulates MT2A gene, whose mRNA transcript isoform appears to be associated with cell proliferation in invasive ductal cancer tissues and that, on the contrary, HIPK2 depletion correlates with MT2A up-regulation in MCF7 breast cancer cells [68]. Moreover, MT2A depletion by siRNA (silencing RNA) in cells depleted of HIPK2, restores wt53 conformation [68].

### HIPK2 inactive/active switch in Alzheimer's disease and the relevance of zinc supplementation

Given the role of HIPK2 in maintaining wt53 conformation in tumor cells and the presence of an unfolded state of p53 in AD peripheral cells, the interest of a putative modulation of HIPK2 in AD type dementia has been investigated. As reported above, the exposure to nanomolar concentrations of Aβ led to an increased content of unfolded p53 protein in fibroblasts from AD patients, compared to control subjects[3]. Looking at the molecular mechanism of p53 misfolding, a link among Aβ, p53 and HIPK2 in the neuropathology may be proposed [73]. Aβ has been hypothesized to be responsible for HIPK2 proteasomal degradation, in turn resulting in HIPK2 nuclear disappearance from target promoters such as hypoxia inducible factor 1α (HIF-1α) [74] and MT2A [68], whose mRNA was found up-regulated in cellular models of AD [73]. The induction of MT2A, depending on HIPK2 knockdown, has been reported to be responsible for p53 misfolding and inhibition of p53 transcriptional activity [68]. On this basis, we could speculate that, in AD, HIPK2 deregulation might be involved in p53 misfolding, most likely through MT2A upregulation.Data from literature point out that MTs play a very important role in controlling Zn^2+^homeostasis. Increased MTs levels induce down-regulation of many biological functions related to Zn^2+^, such as metabolism, gene expression and signal transduction [75].The MTs can serve as a source of Zn^2+^and are considered also as strong anti-oxidants and protective factors against stress conditions [76]. MTs are highly expressed in both astrocytes and hippocampal neurons in the aging brain and are a key area of investigation for inflammation and modulation of Zn^2+^ availability in the aging brain [77]. Therefore these proteins are being intensively studied in the context of aging and longevity mechanisms [78]. During aging there is an increased expression of MTs mRNA but decreased levels are found in healthy centenarians, indicating a possible selection for survival of low expressors [79]. However the precise function of these proteins in aging is still debated, because their protective role could also be deleterious in the case of sequestration of Zn^2+^ as observed both in human aging and AD [73,80,81]. The high MTs mRNA in lymphocytes from old people and Down syndrome subjects and the low MTs mRNA in lymphocytes from young adult and centenarians are a significant support to the idea of a pleiotropic role of MTs in aging [82]. Therefore, the role of HIPK2 in MT2A regulation could unveil interesting interplay between these molecules with p53 also in other chronic diseases such as AD. Taking into account MT2A upregulation dependent on HIPK2 depletion, zinc supplementation to hypoxia-treated cancer cells reestablishes HIPK2 nuclear localization and DNA binding activity, restoring p53 apoptotic function in response to anticancer drug [83]. Similarly, zinc-supplementation to AD cellular models restores HIPK2 DNA binding, p53 wild-type conformation and apoptotic activity in response to a genotoxic agent [73]. Hence, we could define that, in AD, HIPK2 plays a critical role in maintaining p53 wild-type conformation indirectly through MT2A down regulation, and that Zn^2+^ is a fundamental cofactor.

**Figure 2.**
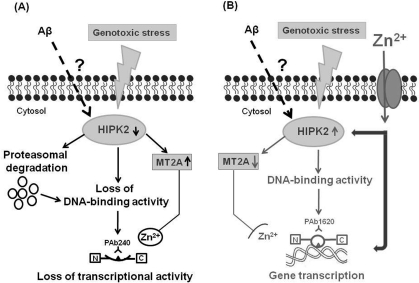
Working hypothesis for a putative link among p53 protein, soluble Aβ and HIPK2. The figure shows a novel mechanism of HIPK2 deregulation mediated by Aβ. HIPK2, when activated in response to DNA damaging agents, is able to interact physically and functionally with p53 and phosphorylate p53 at serine 46, thus regulating p53-induced apoptosis. HIPK2 also acts as transcriptional corepressor and deregulates the promoter metallothionein 2A (MT2A). MT2A may regulate p53 activity inducing protein folding through zinc modulation. In the presence of soluble Aβ, HIPK2 expression and activity are inhibited through Aβ-induced degradation via the proteasome system (panel **A**). HIPK2 deregulation results in the induction of MT2A (panel **A**), that exerts its Zn^2+^ chelator function. As a consequence, p53 protein misfolding (changing the wild-type conformation to a conformationally altered status) with subsequent abolishment of wild type p53 DNA binding and transcriptional activity occurs (panel **A**). Zinc supplementation counteracts Aβ effects on HIPK2 regulation (panel **B**). Zinc enters into cells through specific zinc transporters, that are required to convey this ion across cellular membranes, since zinc is unable to passively diffuse across cell membranes. Zinc can directly restore p53 function (panel **B**). In addition, zinc can also affect HIPK2 function, thus resulting in HIPK2 reactivation (panel **B**). As consequence, MT2A is deregulated and p53 conformational can switch to the wild-type and transcriptional active form (panel **B**).

On the basis of data here summarized, we speculate that soluble Aβ may be responsible for important modulatory effects at cellular level before triggering the amyloidogenic cascade. One of these modulatory effects may be the inhibition of HIPK2 activity, with MT2A upregulation, in turn responsible for the induction of an altered conformational state of p53. As a result of this conformational change, p53 looses its transcriptional activity and is unable to properly activate an apoptotic program when cells are exposed to a noxious stimulus. Altogether, Aβ-induces HIPK2 depletion and the consequent unfolded p53 may contribute to AD pathogenesis leading to dysfunctional cells (Figure 2).

## CONCLUSIONS

In humans, aging may be influenced by the balance of cell survival versus cell death, a decision at least in part regulated by checkpoints proteins, by preservation of DNA integrity and correct repair [84]. We focused mainly on one of such proteins, p53, recently shown to be involved in aging and AD [55,57,58]. A link between AD pathology and an unfolded state of p53 has been proposed, based on findings that with aging an increase of unfolded p53 occurs in healthy subjects and is peculiarly high in AD patients. By investigating what could be the contribution of a conformational change of p53 to AD pathogenesis, for the first time we define a hierarchical scale of events driven by Aβ: Aβ-induced HIPK2 depletion and unfolded p53 may contribute to AD pathogenesis leading to dysfunctional cells [73]. This observation is intriguing in light of recent data showing that p53 suppresses cellular aging. In this context, despite the well-known capability of p53 to induce senescence, more recent evidence demonstrated that p53 can also act as a suppressor of cellular senescence while promoting cell cycle arrest [85]. This dual effect on senescence may be ascribed to the fact that p53 regulates cell growth and metabolic stress through different pathways [86]. One of these is represented by mTOR pathway, which is strictly connected with organismal aging, as its inhibition may be one of the main mechanisms decreasing lifespan [87]. p53 is able to regulate activity of mTOR following DNA damage or oncogenic stress by activation of specific effectors (PTEN, AMP kinase and TSC-2), each of which signals to diminish the activity of mTOR, which is involved in senescent phenotype [85,88]. By suppressing mTOR, p53 can suppress senescent phenotype, converting senescence into quiescence [89]. Furthermore, mTOR inhibition induces autophagy, thus resulting in the accumulation of protein aggregates, endoplasmic reticulum stress and mitochondrial dysfunction, each of which could promote senescence [86]. Thus, taking into account that with aging an increase of unfolded p53 occurs, the loss of wild-type p53 conformation could free mTOR, thus inducing aging-associated abnormalities.

Thirty years have passed since p53 discovery and in these decades a lot of information about its structure, functions and pathways has been achieved. In the fourth decade of p53 investigation the research community hopes to be able to get new drugs to affect p53 function to treat not only cancer but also important neurological conditions, such as AD.The recognition of HIPK2 as new target of the effect of Aβ could suggest a new putative functional biomarker useful in addressing new therapeutic strategies [90].
